# Interactions between Gene Variants within the *COL1A1* and *COL5A1* Genes and Musculoskeletal Injuries in Physically Active Caucasian

**DOI:** 10.3390/genes12071056

**Published:** 2021-07-09

**Authors:** Katarzyna Leźnicka, Ewelina Żyżniewska-Banaszak, Magdalena Gębska, Anna Machoy-Mokrzyńska, Anna Krajewska-Pędzik, Agnieszka Maciejewska-Skrendo, Agata Leońska-Duniec

**Affiliations:** 1 Department of Physical Education, Academy of Physical Education and Sport in Gdansk, ul. Kazimierza Górskiego 1, 80-336 Gdansk, Poland; agata.leonska-duniec@awf.gda.pl; 2 Department of Rehabilitation Musculoskelatal System, Pomeranian University of Medical Science, ul. Unii Lubelskiej 1, 71-252 Szczecin, Poland; banaszak@pum.edu.pl (E.Ż.-B.); mgebska@pum.edu.pl (M.G.); 3 Department of Experimental and Clinical Pharmacology, Pomeranian Medical University, ul. Powstańców Wlkp. 72, 70-111 Szczecin, Poland; amachoy@pum.edu.pl; 4 Faculty of Physical Education and Health, Institute of Physical Education Science, University of Szczecin, al. Piastów 40b, 70-237 Szczecin, Poland; anna.krajewska-pedzik@usz.edu.pl (A.K.-P.); maciejewska.us@wp.pl (A.M.-S.)

**Keywords:** injury and prevention, physical activity, polymorphisms, collagen, musculoskeletal, sport genetics

## Abstract

The *COL1A1* and *COL5A1* variants have been associated with the risk of musculoskeletal injuries. Therefore, the main aim of the study was to investigate the association between three polymorphisms within two genes (rs1800012 in *COL1A1*, as well as rs12722 and rs13946 in *COL5A1*) and the reported, yet rarely described in the literature, injuries of the joint and muscle area in a physically active Caucasian population. Polish students (*n* = 114) were recruited and divided into the following two groups: students with (*n* = 53) and without (*n* = 61) injures. Genotyping was carried out using real-time PCR. The results obtained revealed a statistically significant association between rs1800012 *COL1A1* and injury under an overdominant model. Specifically, when adjusted for age and sex, the GT heterozygotes had a 2.2 times higher chance of being injured compared with both homozygotes (TT and GG, 95% CI 0.59–5.07, *p* = 0.040). However, no significant interaction between the *COL5A1* variants, either individually or in haplotype combination, and susceptibility to injury were found. In addition, the gene–gene interaction analysis did not reveal important relationships with the musculoskeletal injury status. It was demonstrated that rs1800012 *COL1A1* may be positively associated with physical activity-related injuries in a Caucasian population. Harboring the specific GT genotype may be linked to a higher risk of being injured.

## 1. Introduction

Physical activity promotion has become an important element of public health due to the sedentary lifestyle and high caloric intake of the global population resulting in an obesity epidemic [1]. Regular exercise has numerous benefits for human health and well-being, including a reduction of the numerous chronic diseases, such as cardiovascular diseases, diabetes, and various forms of cancer; and an improvement of mental health. Moreover, it contributes to improved body composition parameters and helps to control weight [2,3]. However, the promotion of training programs may enhance the risk of physical activity-related injuries (PARIs), as they most frequently occur within the physically active group of people [4,5,6].

Participation in professional sports training or recreational physical activity can cause acute and chronic injuries of the musculoskeletal system, including tendons, ligaments, and skeletal muscles. The background of the aforementioned injuries remains mostly unknown, with several risk factors being proposed. Initially, many studies performed in families, adoptees, and twins showed a genetic contribution to interindividual variation in the human body’s reaction to mechanical load, thus influencing the integrity of the tissue, its function, and finally injury risk [7]. For athletes, training and competition time lost to injuries has a profoundly negative impact on their performance. Therefore, most research on the genetic determinants of injuries has focused on athletes who are professionally practicing sports while considerably less research has focused on the population of physically active women and men [8,9]. It is important to identify associations between environmental factors, genetic markers, and musculoskeletal injuries and as a consequence predict the possible risk of these injuries for physically active people and guide the clinical management of “high-risk” individuals [10,11].

To date, more than 70 genes and chromosomal regions have been investigated regarding their associations with musculoskeletal soft tissue injuries, such as: Achilles tendon pathology (ATP), Achilles tendinopathy (AT), Achilles tendon ruptures (ATRs), shoulder dislocations (SDs), tennis elbow (TE), and anterior cruciate ligament rupture (ACLR) [7,8,12,13]. Among these candidate loci, distinguished genes encode a wide spectrum of matrix proteins, including structural components, such as collagens, as well as proteoglycans, cytokines, matrix proteinases, and signaling and apoptosis factors [7]. The injured susceptibility gene that was first described was the collagen type I alpha 1 chain gene (*COL1A1*), which was positively associated with ACLR in athletic and non-athletic cohorts [8,14,15,16]. Since then, several genes encoding collagens have been shown to be associated with the potential risk of connective tissue disorders and/or musculoskeletal injuries [7].

Within the human *COL1A1* gene, localized in chromosome region 17q21.33, single nucleotide polymorphisms (SNPs) have been described, which may alter *COL1A1* expression and consequently affect the collagen type I properties and lead to a susceptibility to injuries. One of the most frequently studied genetic variants is the functional SNP with a G to T change lying within the first intron of the gene, thus affecting a binding site for the transcription factor Sp1 (*COL1A1* Sp1 + 1245G/T, rs1800012). This variant was initially described in 1996 [9,17]. A lower frequency of the TT genotype among participants with ACLR has been described, implying that some individuals may benefit from carrying the T allele [10].

The second injury susceptibility gene is the collagen type V alpha 1 chain gene (*COL5A1*), localized to the long arm of chromosome 9 (9q34.3), with its product playing an important role in regulating the fiber diameter as well as the assembly (fibrillogenesis) of collagen fibers. Polymorphisms within the *COL5A1* gene have been shown to impact the classic form of Ehlers–Danlos syndrome, which is characterized by joint hyper mobility [18] and other joint dysfunctions [19], as well as ACLR [20]. The most considerable polymorphisms are rs12722 (BstUI) and rs13946 (DpnII), which are localized in the 3′-untranslated region (UTR). A higher frequency of the *COL5A1* C-T (rs12722-rs13946) haplotype was associated with a reduced risk of ACLR in a group of Polish male recreational skiers [20].

The results of the aforementioned studies suggest that there are significant differences in genetic profiles that predispose individuals to musculoskeletal injuries. The most common PARIs for which a genetic contribution has been observed include AT in the heel, damage to the rotator cuff in the shoulder, and the rapture of cruciate ligaments in the knee [21]. A small number of available scientific reports have described the relationship between genetic profiles and less common injuries in joints and muscles, such as muscle tears, sprains, and complete and partial fractures, as well as joint capsule damage. Moreover, in the published studies, the presented results concern injuries in groups of athletes, often professionals, who had particular injuries significantly more often than non-athletes (ACLR, AT). Increasing the number of genetic tests and determining their relationship with injuries will allow scientists to estimate, with high probability, which genetic variants predispose to acute damage of the musculoskeletal system. Therefore, the main aim of our study was to evaluate the association between three polymorphisms within two genes (rs1800012 in *COL1A1*, as well as rs12722 and rs13946 in *COL5A1*) and reported motor organ injuries in physically active young people who do not practice any sport professionally.

## 2. Materials and Methods

The experimental process included the following steps: obtaining consent for the examination, recruitment of volunteers, collection of genetic material, genetic isolation, genotyping, statistical analysis, and the description of the results.

### 2.1. Ethics Statement

The Pomeranian Medical University Ethics Committee (Szczecin, Poland) verified the compliance of the investigation protocols (KB-0012/104/15). The procedures were conducted ethically according to the World Medical Association Declaration of Helsinki and to the Strengthening the Reporting of Genetic Association studies statement (STREGA). All participants were informed of the risks and benefits of the experiment and gave written consent to genotyping. All personal information and results were anonymous.

### 2.2. Participants

In total, 114 students aged M = 23.14 (SD = 5.14) years, from the Szczecin University and Pomeranian Medical University participated in the study. The study included those students who declared systematic participation in physical activity two or more times a week. The participants were asked to provide detailed information on the trauma, such as its location and type (sprain, fracture, discopathy, damage to the joint capsule, muscle tear, ligament rupture). The classification of the individuals into those with (*n* = 53) and without (*n* = 61) these injuries was based on their declaration as regards motor organ system injuries experienced in the preceding 5 years. 

Anthropometric data were obtained based on a single measurement performed directly before the study. Body height was measured with an anthropometer (Vitako, Warszawa, Poland) whereas body weight was measured with an electronic scales (Radwag, Radom, Poland), with an accuracy of 1 cm and 0.1 g, respectively.

### 2.3. Genotyping

DNA was extracted from the buccal cells using a GenElute Mammalian Genomic DNA Miniprep Kit (Sigma, Steinheim, Germany) according to the manufacturer’s protocol. All samples were genotyped in duplicate. An allelic discrimination assay was used on a C1000 Touch Thermal Cycler (Bio-Rad, Feldkirchen, Germany) instrument with TaqMan^®^ probes. To discriminate rs1800012 (COL1A1), rs13946 (COL5A1), and rs12722 (COL5A1) alleles, TaqMan^®^ Pre-Designed SNP Genotyping Assays were used (Applied Biosystems, Waltham, MA, USA) (assay ID: C___7477170_30, C___8721600_10, and C____370252_20, respectively), containing fluorescently labelled (FAM and VIC) minor groove binder (MGB) probes and primers.

### 2.4. Statistical Analysis

Statistical analysis was performed in R (https://cran-r.project.org (accessed on 23 April 2018) using generalized linear models. For models evaluating the main genotype effects, we used three modes of penetrance: dominant, recessive, and overdominant, which were constructed with respect to the minor allele. For models with interaction terms, in addition to dominant (at least one minor allele in both loci) and recessive (rare homozygous genotypes in both loci), a homozygote-heterozygote (HOM-HET) model was evaluated. A general HOM-HET model was further broken down into four following variants: HOM1-HET, HOM2-HET, HET-HOM1, and HET-HOM2, where HOM1 corresponds to the rare (minor) allele homozygous genotype and HOM2 to common allele homozygotes. Age and sex were used as covariates in all models. *p* value < 0.05 was considered significant.

For multiple-hypothesis testing error, the false discovery rate (FDR) was controlled using the Benjamini and Hochberg method within genes (gene–gene interaction space).

For a single gene analysis, the estimated power for the dominant model ranged from 6% (OR 1.1) to 45% (OR 2.0) for the following assumptions: risk of injury 15–20–25–30%, minor allele frequency 20–30%, and odds ratio 1.1–2.0. For a gene–gene interaction, an estimated power for the dominant model and the same assumptions ranged from 5% (OR 1.1) to 14% (OR 2.0). The estimated power for the recessive models was reduced by about half. Power analysis was conducted using Quanto (version 1.2.4, Gauderman and Morrison [22].

## 3. Results

### 3.1. Genotypes Analysis

Genotype, allelic frequencies, and Hardy–Weinberg equilibrium (HWE) *p* values are shown in Table 1. Except for the *COL5A1* rs13946 genotype frequencies, which conformed to Hardy–Weinberg equilibrium, the observed frequencies deviated significantly from expectations (*p* < 0.001) respectively, for *COL1A1* rs1800012 and *COL5A1* rs12722).

In Table 2, Table 3 and Table 4, the results of an association between the *COL1A1* and *COL5A1* variants and injury status with and without adjustment for age and sex are shown. We found a significant association between rs1800012 *COL1A1* and injury under the overdominant model. Specifically, when adjusted for age and sex, GT heterozygotes had a 2.2 times higher chance of being injured compared with both homozygotes (TT and GG, 95% CI 0.59–5.07, *p* = 0.040, FDR = 1.200). No significant associations were found when other underlying genetic models were assumed (Table 2). Similarly, no significant associations between the *COL5A1* variants and susceptibility to injury were found regardless of the assumed underlying genetic models (Table 3 and Table 4).

### 3.2. Haplotype Characteristics of Our Research Group

Haplotype-based analysis based on unphased genotypes revealed four *COL5A1* NM_000093.4:c [rs13946;rs12722] haplotypes: one common haplotype T-T with one frequency of 58.5%, a C-C haplotype with a frequency of 23.9%, and two remaining C-T and T-C haplotypes with frequencies of about 9%. Haplotype-based association analysis (Table 5) revealed no significant relationships regardless of the model of haplotype inheritance. We also investigated gene–gene interaction assuming three major models: dominant, recessive, and homozygote-heterozygote (HOM-HET), and four more variants of the latter (Table 6). However, no significant epistatic interactions for injury susceptibility were observed. 

## 4. Discussion

Many studies show a worrying increase in the incidence of physical activity-related injuries. Almost half of all adolescents participating in sports club activities have reported at least one PARI in the past year [23]. From a public health perspective, there is a pressing need to invest in injury prevention to reverse this trend.

The aim of our study was to examine the association between the COL1A1 and COL5A1 variants, and their interaction with the musculoskeletal injury status with and without adjustments for age and sex. The main finding of the data was a statistically significant association between the rs1800012 COL1A1 with injuries under the overdominant model. A detailed analysis showed that the GT heterozygotes had a 2.2 times higher risk of being injured compared to TT and GG homozygotes, suggesting that this specific genotype may be a risk factor involved in PARIs in the Caucasian population. Conversely, some individuals may benefit from carrying the TT and GG genotypes. Due to the value of the FDR, the interpretation of the results should be approached with caution. This relationship was significant both in women and men. However, our analysis of the association between both the rs12722 and rs13946 polymorphisms in the COL5A1 gene, either individually or in haplotype combination, and musculoskeletal injury showed no significant differences in genotype or allele distribution between the group with and without PARI. Additionally, the gene–gene interaction analysis revealed no significant relationships with the musculoskeletal injury status regardless of the statistical model used.

Recently, many studies have indicated SNPs in the COL1A1 gene, which have been associated with an increased risk of several complex connective tissue disorders, such as SD, ATR, AT, and ACLR [24,25,26]. It was shown that the TT genotype of rs1800012 COL1A1 has a possible preventive role not only in ACLR but also in other soft tissue injuries. According to the authors, the TT genotype was associated with about a 10 times lower incidence of soft tissue injuries (cruciate rupture, SD, and bone mineral density of ATR) in all studied athletes and patients compared to the control groups [14,27]. Another study conducted on the Polish Caucasian population investigated whether the COL1A1 rs1107946 and rs1800012 polymorphisms, individually and as haplotypes, influence the risk of ACLR in a group of professional soccer players. The authors revealed that a higher frequency of the COL1A1 G-T (rs1107946-rs1800012) haplotype is associated with a lower risk of this injury. A protective effect of carrying two copies of this particular haplotype against ACLR was suggested. Although they found no statistically significant differences in the genotype distribution of the COL1A1 SNPs and the ACLR when analyzed individually, it was highlighted that none of the participants with ACLR carried the TT genotype (rs1800012) [8]. A possible functional explanation for these observations was provided by Mann et al., who proposed COL1A1 Sp1 (rs1800012) as a functional polymorphism that influences Sp1 binding and gene regulation, increasing the collagen alpha1(I) chain production relative to alpha2(I) and reducing bone strength [28]. However, Urreizti et al. did not find any association between the COL1A1 (rs1800012) genotype and fracture risk in Spanish individuals [29]. In a study including Polish male recreational skiers, Stępień-Słodkowska et al. found the opposite results, indicating that the ACLR risk was around 1.43 times lower in G allele carriers as compared to T allele carriers [30]. The present study’s findings connect the results of Ficek et al. and Stępień-Słodkowska et al. obtained on a Caucasian population, suggesting that PARIs may occur more frequently in people with the GT genotype compared to homozygotes, indicating that the TT and GG genotype may have a protective effect against PARIs. However, more experimental studies are needed to establish the interaction between SNPs within the COL1A1 gene and the risk of musculoskeletal injury [8,30].

Compared to collagen type I, quantitatively, collagen type V is forms less fibers. There is evidence to suggest that it is functionally the main collagen in developing connective tissues [31]. An analysis of 9 studies involving 1140 cases and 1410 healthy controls indicated that rs12722 COL5A1 was positively connected with tendon and ligament injuries, especially in a Caucasian population. Individuals with the TT genotype were predisposed to a higher risk of ATP, ACLR, and TE [32]. Other studies confirmed a highly significant association between the rs12722 polymorphism, but not the rs13946, and symptomatic chronic AT in a South African population and Australian participants of white ancestry. In addition, these studies showed that individuals with the CC genotype rs12722 had a significantly decreased risk of developing chronic AT compared with those with the T allele (TC or TT genotype) in both populations [33,34]. These two SNPs are localized in the 3′-UTR region and they probably influence the mRNA stability and its post-transcriptional export from the nucleus, where regulatory sequences affect the expression of the gene [35]. As a result, genetic variants within this gene region may alter the secondary structure of mRNA and thus protein features [36]. Thus, these SNPs are believed to modify the stability of the COL5A1 mRNA [35]. However, our analysis of the association between both the rs12722 and the rs13946 polymorphisms in the COL5A1 gene and musculoskeletal injury showed no significant differences in the genotype or allele distribution between the group with PARIs and the control group. The lack of significant differences in our study of both polymorphisms, either individually or in haplotype combination, may be caused by an insufficient number of cases of ACLR (only five cases reported) and a complete absence of AT or Achilles rupture. Another study conducted on the Polish Caucasian population by Stępień-Słodkowska et al. also showed no statistically significant differences in the allele and genotype distribution of the COL5A1 polymorphisms. However, they noticed that the COL5A1 C-T (rs12722-rs13946) haplotype is associated with a decreased risk of ACLR in a group of Polish male recreational skiers [20].

A potential limitation of this study was that the size of the study cohort was too small, which might not have displayed statistical power sufficient to allow meaningful analysis and interpretation of the results obtained. Additionally, in the group of students with PARIs, more than half of the reported injuries concerned soft tissue damage in the joint area, while the rest concerned bone injuries (fractures), which made it impossible to perform a full analysis taking into account individual types of injuries. It needs to be highlighted that genetic marker analysis may be useful for the development of performance tests, making training programs more efficient and safer. The genetic tests may be applied for pre-participation risk screening and may prevent sudden incidents during sport. Physicians, trainers, and therapists should be aware that genetic factors may play a key role in determining musculoskeletal injury risk and the response to therapeutic interventions. However, understanding the exact role of the genetic markers in these aspects requires further research.

## 5. Conclusions

The results of our experiment suggest that rs1800012 COL1A1 may be positively associated with musculoskeletal injury in a Caucasian population. It was demonstrated that harboring the specific GT genotype is linked to a 2.2 times higher risk of being injured, suggesting that this specific genotype is a risk factor involved in PARIs in the studied population. Conversely, some individuals may benefit from carrying the TT and GG genotypes. This relationship was significant both in women and men. However, our analysis of the association between both the rs12722 and rs13946 polymorphisms in the COL5A1 gene and musculoskeletal injuries showed no significant differences in the genotype or allele distribution between the group with PARIs and the control group. In addition, the haplotype-based and the gene–gene interaction analysis revealed no significant relationships with the injury status.

## Figures and Tables

**Table 1:** Genotype and allele frequencies of the *COL1A1* and *COL5A1* polymorphisms.

SNP	Genotype Frequency	Allele Frequency	*p* (HWE *)
*COL1A1* rs1800012	GG 75 (65.8%)	G 172 (75.4%)	<0.001
	GT 22 (19.3%)	T 56 (24.6%)	
	TT 17 (14.9%)		
*COL5A1* rs12722	TT 63 (55.3%)	C 74 (32.5%)	<0.001
	TC 28 (24.6%)	T 154 (67.5%)	
	CC 23 (20.2%)		
*COL5A1* rs13946	TT 54 (47.4%)	T 154 (67.5%)	0.397
	TC 46 (40.3%)	C 74 (32.5%)	
	CC 14 (12.3%)		

* HWE-Hardy-Weinberg equilibrium.

**Table 2:** Association of the rs1800012 *COL1A1* polymorphism with injury status.

Model	Injury (Yes) (*n* = 53)	Injury (No) (*n* = 61)	OR (95% CI)	*p*
	Dominant			
GG	32 (42.2%)	43 (55.8%)	0.64 (0.29–1.39)	0.258/0.286 *
GT-TT	21 (54.9%)	18 (45.1%)	1	
	Recessive			
GT-GG	45 (48.8%)	52 (51.2%)	0.97 (0.34–2.80)	0.959/0.919 *
TT	8 (42.9%)	9 (42.9%)	1	
	Overdominant			
TT-GG	40 (44.0%)	52 (56.0%)	1.88 (0.74–4.97)	0.058/0.040 *
GT	13 (63.3%)	9 (36.7%)	1	

* Age and sex adjusted.

**Table 3:** Association of the rs12722 *COL5A1* polymorphism with injury status.

Model	Injury (Yes) (*n* = 53)	Injury (No) (*n* = 61)	OR (95% CI)	*p*
	Dominant			
TT	32 (48.0%)	31 (52.1%)	1.47 (0.70–3.13)	0.307/0.354 *
TC-CC	21 (37.3%)	30 (62.7%)	1	
	Recessive			
TT-TC	44 (44.4%)	47 (55.6%)	1.46 (0.58–3.82)	0.430/0.573 *
CC	9 (37.5%)	14 (62.5%)	1	
	Overdominant			
CC-TT	41 (45.4%)	45 (54.6%)	0.82 (0.34–1.94)	0.657/0.571
TC	12 (37.1%)	16 (62.9%)	1	

* Age and sex adjusted.

**Table 4:** Association of the rs13946 *COL5A1* polymorphism with injury status.

Model	Injury (Yes) (*n* = 53)	Injury (No) (*n* = 61)	OR (95% CI)	*p*
	Dominant			
TT	26 (50.0%)	28 (50.0%)	0.14 (0.54–2.38)	0.737/0.938 *
TC-CC	27 (44.1%)	33 (56.0%)	1	
	Recessive			
TT-TC	46 (47.1%)	54 (52.9%)	0.85 (0.27–2.66)	0.779/0.859 *
CC	7 (45.0%)	7 (55.0%)	1	
	Overdominant			
CC-TT	33 (48.9%)	35 (51.1%)	0.81 (0.38–1.73)	0.596/0.842 *
TC	20 (43.8)	26 (56.3%)	1	

* Age and sex adjusted.

**Table 5:** The *COL5A1* NM_000093.4:c.[rs13946-rs12722] association with injury status.

Haplotype		DOM		REC		ADD	
		β (SE)	*p* †	β (SE)	*p*	β (SE)	*p* †
*COL5A1* NM_000093.4:c.[rs13946-rs12722]	H1 [C-C]	−0.38 (0.43)	0.368/ 0.431	0.16 (0.57)	0.778/ 0.847	−0.13 (0.29)	0.648/ 0.723
	H2 [C-T]	0.30 (0.55)	0.578/ 0.343	---	---	0.39 (0.55)	0.468/ 0.268
	H3 [T-C]	−0.47 (0.57)	0.410/ 0.645			−0.34 (0.48)	0.479/ 0.774

† without and with age and sex adjustment.

**Table 6:** Association of the *COL1A1* × *COL5A1* interaction with injury status.

SNP1	SNP2	Model OR (95% CI), *p*						
		DOM	REC	HOM-HET	HOM1-HET	HOM2-HET	HET-HOM1	HET-HOM2
*COL1A1* rs1800012	*COL5A1* rs13946	2.09 (0.70–6.76) 0.188 †	-- ^1^	1.01 (0.47–2.17) 0.984 †	AA-TC 1.49 (0.37–6.44) 0.574 †	CC-TC 0.61 (0.24–1.48) 0.276 †	-- ^2^	CA-TT 1.72 (0.56–5.59) 0.341 †
*COL1A1* rs1800012	*COL5A1* rs12722	2.05 (0.62–7.47) 0.243 †	1.17 (0.13--10.58) 0.884 †	0.79 (0.36–1.75) 0.569 †	AA-TC 1.87 (0.30–14.37) 0.430 †	CC-TC 0.40 (0.13–1.14) 0.086 †	-- ^3^	CA-TT 1.79 (0.67–5.05) 0.248 †

^1^ only one CA-TC compound genotype (in the injury group), ^2^ no CA-CC (HET-HOM1) compound genotype (in the injury group), ^3^—only two CA-CC compound genotypes (in the no-injury group), † age- and sex-adjusted *p* values.

## Data Availability

The data presented in this study are available on request from the corresponding author.
